# P41 Use of delafloxacin in osteomyelitis: a case report

**DOI:** 10.1093/jacamr/dlad066.045

**Published:** 2023-06-14

**Authors:** Madhuri Vidwans, Andra Mitria, Hala Kandil

**Affiliations:** West Hertfordshire Teaching Hospitals NHS Trust, UK; West Hertfordshire Teaching Hospitals NHS Trust, UK; West Hertfordshire Teaching Hospitals NHS Trust, UK; Faculty of Medicine, Alexandria University, Egypt

## Abstract

**Background:**

Data and experience with delafloxacin use in osteomyelitis (OM) and other deep-seated infections are limited, yet the burden of OM on healthcare systems is significant. We hereby describe the first case, to our knowledge, of delafloxacin use in OM.

**Case description:**

A 94-year-old male with a history of prostate cancer, hypertension and heart failure was admitted to hospital in June 2022 with grade 4 bilateral heel ulcers. Empirical IV co-amoxiclav was started and then escalated to IV piperacillin/tazobactam. MRI scans confirmed the diagnosis of OM, and tissue and bone samples grew MDR *Pseudomonas aeruginosa* (piperacillin/tazobactam-resistant and ciprofloxacin intermediate), an additional challenge. Due to this result and poor clinical response, IV meropenem was prescribed. Off-label use of delafloxacin was discussed within the multidisciplinary team (MDT) to switch to oral administration, decrease length of hospital stay and account for the patient’s age and co-morbidities. Minimum inhibitory concentration of the MDR *P. aeruginosa* using EUCAST guidelines demonstrated delafloxacin susceptibility (0.125 mg/L). Delafloxacin also has good bioavailability and does not require therapeutic drug monitoring. The patient was started on 450 mg delafloxacin twice daily with monitoring of clinical response. After 10 days of initial in-hospital clinical monitoring, the patient was discharged home with 450 mg twice daily delafloxacin to complete 28 days of therapy. Follow-up imaging was not planned given his age and co-morbidities. District nurses reported improvement in his heel ulcers. On follow-up telephone consultation with the treating consultant in November 2022, the patient reported feeling well and noted improvement in his ulcers. Readmission and adverse events have not been reported to date and the ulcers continue to improve.

**Conclusions:**

This case presents the first successful use of delafloxacin in osteomyelitis, with good tolerability and clinical response against MDR *P. aeruginosa*. Delafloxacin enabled our patient to be discharged 3 weeks earlier than planned which would otherwise not have been possible, improving his quality of life and reducing risk of hospital-acquired infections with associated costs. Delafloxacin also enables early IV to oral switch in support of the antimicrobial stewardship agenda.

